# Diagnostic Values of the Liver Imaging Reporting and Data System in the Detection and Characterization of Hepatocellular Carcinoma: A Systematic Review and Meta-Analysis

**DOI:** 10.7759/cureus.36082

**Published:** 2023-03-13

**Authors:** Arvin Arian, Ayoob Dinar Abdullah, Hayder J Taher, Hayder Suhail Alareer, Maryam Fotouhi

**Affiliations:** 1 Department of Radiology, Cancer Institute-Advanced Diagnostic and Interventional Radiology Research Center (ADIR) Tehran University of Medical Sciences (TUMS), Tehran, IRN; 2 Department of Technology of Radiology and Radiotherapy, Tehran University of Medical Sciences (TUMS), Tehran, IRN; 3 Department of Radiology, Hilla University College, Babylon, IRQ; 4 Department of Technology of Radiology and Radiotherapy, International Campus, Tehran University of Medical Sciences (TUMS), Tehran, IRN; 5 Quantitative MR Imaging and Spectroscopy Group (QMISG), Research Center for Molecular and Cellular Imaging (RCMCI) Advanced Medical Technologies and Equipment Institute (AMTEI) Tehran University of Medical Sciences (TUMS), Tehran, IRN

**Keywords:** meta-analysis, systematic review, hepatocellular carcinoma, li-rads, liver imaging

## Abstract

This review was undertaken to assess the diagnostic value of the Liver Imaging Reporting and Data System (LI-RADS) in patients with a high risk of hepatocellular carcinoma (HCC). Google Scholar, PubMed, Web of Science, Embase, PROQUEST, and Cochrane Library, as the international databases, were searched with appropriate keywords.

Using the binomial distribution formula, the variance of all studies was calculated, and using Stata version 16 (StataCorp LLC, College Station, TX, USA), the obtained data were analyzed. Using a random-effect meta-analysis approach, we determined the pooled sensitivity and specificity. Utilizing the funnel plot and Begg’s and Egger’s tests, we assessed publication bias.

The results exhibited pooled sensitivity and pooled specificity of 0.80% and 0.89%, respectively, with a 95% confidence interval (CI) of 0.76-0.84 and 0.87-0.92, respectively. The 2018 version of LI-RADS showed the greatest sensitivity (0.83%; 95% CI 0.79-0.87; *I*^2^ = 80.6%; *P* < 0.001 for heterogeneity; *T*^2 ^= 0.001). The maximum pooled specificity was detected in LI-RADS version 2014 (American College of Radiology, Reston, VA, USA; 93.0%; 95% CI 89.0-96.0; *I*^2 ^= 81.7%; *P* < 0.001 for heterogeneity; *T*^2^ = 0.001). In this review, the results of estimated sensitivity and specificity were satisfactory. Therefore, this strategy can serve as an appropriate tool for identifying HCC.

## Introduction and background

Hepatocellular carcinoma (HCC), also known as hepatoma, remains the fifth most frequent type of and the second major reason for death from cancer worldwide. Each year, the estimated number of new cases of HCC is about one million, and virtually, 600,000 people die from this cancer [1]. In addition to alcoholic liver disease, infections with hepatitis viruses B (HBV) or C (HCV) have been suggested as the most widespread HCC risk factors [2]. The overall HCC incidence in cirrhosis patients for five years has been estimated to be between 5% and 30%, and the majority of HCC patients (80%-90%) are associated with cirrhosis [3,4].

Considering that obesity and other metabolic syndromes are increasing in the global population, an increased prevalence of HCC, owing to nonalcoholic fatty liver disease, is predictable [5]. Similarly, despite HBV and HCV prevention through vaccination and antiviral treatments, the global incidence of HCC is increasing [6]. The approved system by the American College of Radiology (ACR), Reston, VA, USA, namely, the Liver Imaging Data and Reporting System (LI-RADS), offers a standard for imaging HCC in terms of screening and diagnosis of treatment response, as well as its assessment [7]. This system was designed by a group of radiologists as well as by some expert specialists in liver cancer imaging who developed LI-RADS. LI-RADS was merged with the most recent clinical practice guidelines for HCC by the American Association for the Study of Liver Diseases (AASLD) [7].

Four imaging algorithms covered by LI-RADS comprise ultrasound (US), magnetic resonance imaging (MRI), contrast-enhanced ultrasound (CEUS), and computed tomography (CT). The first method is used for HCC surveillance, while the other three strategies are applied for HCC diagnosis. CT/MRI is also utilized for assessing treatment response to HCC and tumor staging [8]. An algorithm is supported by a categorization table and a decision tree, which help radiologists evaluate the potential for carcinoma based on imaging characteristics. A technique and lexicon that support an algorithm offer standardized terminology and provide the least technical requirements. In accordance with the guidelines offered by the AASLD, a management section offers workup advice [9]. A reporting section helps the radiologist clearly and succinctly communicate relevant information. A comprehensive manual provided by LI-RADS is currently being reviewed by liver imaging specialists. This manual consists of educational materials as well as schematic graphs, diagrams, and clinical instances [8,10]. In this meta-analysis, we evaluated the diagnostic value of LI-RADS in detecting and characterizing HCC.

## Review

Literature search strategy

Google Scholar, PubMed, Web of Science, Embase, PROQUEST, and Cochrane Library, as the international databases, were searched independently on October 20, 2022, by two authors. The following keywords and their combinations, abbreviations, and MeSH terms were used for a systematic search: "hepatocellular carcinoma," "hepatocellular adenoma," "Dynamic Contrast-Enhanced Magnetic Resonance Imaging (DCE-MRI)," "diagnostic," "malignant," and "benign."

Study selection

The following criteria were used to include relevant studies in this review: (1) original articles, (2) published in English, (3) using DCE-MRI as the imaging modality, (4) reporting the diagnostic accuracy, and (5) differentiating HCA or HCC from other hepatocellular tumors. Also, the articles that met the following criteria were excluded: (1) review articles, book chapters, case reports, and letters to the editor; (2) using other imaging modalities except for DCE-MRI; and (3) not using a reference standard.

Screening and data extraction

Before screening the titles and abstracts by two independent authors, duplicate articles were removed. Then, relevant abstracts were considered for full-text review. The identified studies were assessed, and if there was any disagreement, a third author would assess that study. Then, the data from the included articles were extracted. Name of the first author, type and location of study, year of publication, sample size, age, gender, MRI finding, sensitivity, specificity, positive predictive value (PPV), negative predictive value (NPV), and area under the curve (AUC) were all data gathered from included investigations.

Quality assessment

The quality assessment of eligible studies was accomplished by one of the authors by applying the Quality Assessment of Diagnostic Accuracy Studies (QUADAS) criteria, which is a quality assessment tool to evaluate the risk of bias as well as the applicability of initial diagnostic accuracy studies in systematic reviews. Using the QUADAS questionnaire, we assessed the quality of the mentioned investigations in four fields: (1) patient selection, (2) index test(s), (3) reference standard, and (4) flow and timing.

Statistical analysis

The pooled sensitivity, pooled specificity, accuracy, PPV, NPV, and AUC and their 95% CI for HCC and HCA diagnosis were calculated using the Stata statistical software package (version 1; Stata Corp., College Station, TX, USA). Also, bias in the publications was assessed using Begg and Egger’s method. The heterogeneity of each group was measured using the inconsistency index (*I*^2^). Significant heterogeneity is defined as *I*^2^ > 50% or *P* < 0.05. If the heterogeneity was high, a random-effect model was used to calculate the pooling effect and 95% confidence interval (CI). Otherwise, the fixed effect was used. The performance of DCE-MRI and its features for the diagnosis of HCC and HCA, among other hepatocellular tumors, were determined by calculating pooled specificity, sensitivity, accuracy, PPV, NPV, and AUC with 95% CI.

Results

Study Selection

**Figure 1:**
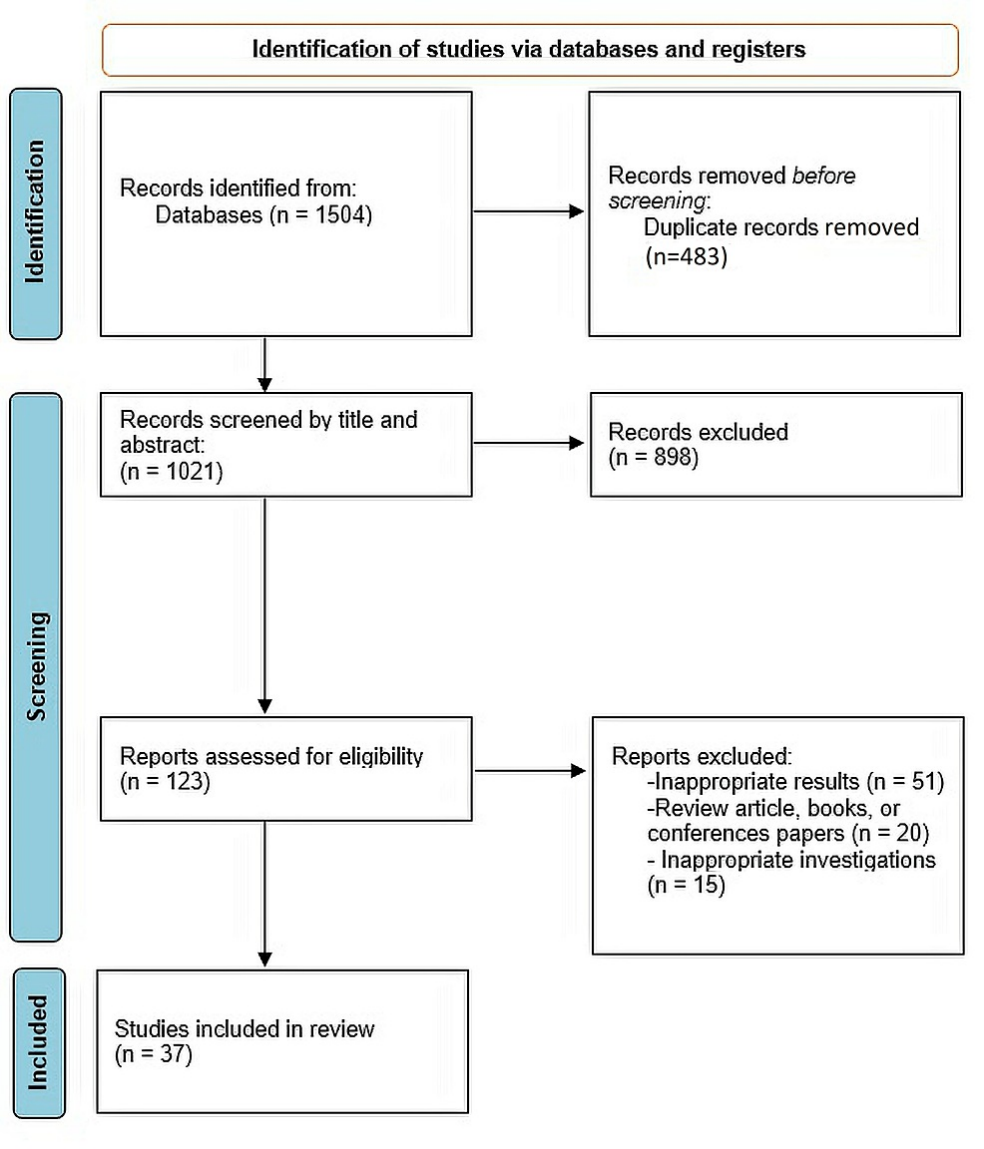
PRISMA protocol. PRISMA, Preferred Reporting Items for Systematic Reviews and Meta-Analyses

The selection process is shown in a flowchart (Figure 1).

After an initial search, 1,504 studies were identified. Four hundred eighty-three duplicate articles were removed, and 1,021 studies were considered for screening. After title/abstract and full-text screening, 898 and 87 studies were excluded, respectively. Finally, 36 studies were eligible for our research.

Features of eligible studies

Table 1 represents the features of the studies selected (*n* = 36). The included articles, consisting of 5,477 patients, reported data about the diagnostic performance of DCE-MRI and its features to differentiate HCC and HCA from other hepatocellular tumors. From the 36 studies, 29 studies enrolled patients retrospectively and the remaining seven articles enrolled patients prospectively.

**Table 1:** Features of included investigations. MRI, magnetic resonance imaging; HCC, hepatocellular carcinoma; CE, contrast-enhanced; DCE, dynamic contrast-enhanced; Gd-EOB, gadolinium ethoxybenzyl; HAP, hepatic arterial phase; HBP, hepatobiliary phase

Author (reference)	Year	Number of patients	Age (mean ± SD) or mean (range)	Male/Female	Type of study	Diagnosis	Imaging modality	MRI features
Hwang et al. [11]	2019	177	58 (32-80)	142/35	Retrospective	HCC	Gadoxetic acid-enhanced MRI	-
Chen et al. [12]	2020	167	49.5 ± 10.3	150/17	Retrospective	HCC	Extracellular CE MRI	-
Cannella et al. [13]	2020	155	57.2 ± 10.6	108/47	Retrospective	HCC	Gadoxetic acid-enhanced MRI	-Subthreshold growth -Fat sparing in solid mass -Restricted diffusion -Mild-moderate T2 hyperintensity -Iron sparing in solid mass -Transitional phase hypointensity -Hepatobiliary phase hypointensity -Nonenhancing capsule -Nodule-in-nodule architecture -Mosaic architecture -Fat in mass, more than adjacent liver -Blood products in mass
Hwang et al. [14]	2020	110	58 (30-89)	79/31	Retrospective	HCC	Gadoxetic acid-enhanced MRI	-Vascular hallmark -Co-HCC ≥ 1cm -Arterial-phase hyperenhancement -Hypointensity on either portal venous phase or transitional phase
Byun et al. [15]	2020	400	59.7 (33-86)	322/78	Retrospective	HCC	Gadoxetic acid-enhanced MRI	-
Ichikawa et al. [16]	2020	269	67.4 ± 10.0	198/71	Retrospective	HCC	Gadoxetic acid-enhanced MRI	-
Stocker et al. [17]	2018	108	41.5 ± 18.3	46/62	Retrospective	HCC	DCE-MRI	-fs-T1w native -Arterial phase hyperenhancement -Portal-venous phase -Hepatobiliary phase
Michallek et al. [18]	2022	63	41 ± 12	16/47	Retrospective	HCC	DCE-MRI	-
An et al. [19]	2019	217	59 (36-85)	166/51	Retrospective	HCC	Gadoxetic acid-enhanced MRI	-
Bashir et al. [20]	2012	100	57.9 (29-91)	57/43	Retrospective	HCC	Gadoxetic acid-enhanced MRI	-
Cha et al. [21]	2017	421	57 (20-82)	303/118	Retrospective	HCC	Gadoxetic acid-enhanced MRI	-
Chen et al. [22]	2015	139	68 ± 11	61/38	Retrospective	HCC	Gadoxetic acid-enhanced MRI	-
Fischer et al. [23]	2015	107	45 ± 14	46/61	Retrospective	HCC	DCE-MRI	-T1 hypointense -T2 hypo- or hyperintense -Lack of central enhancement -Presence of satellite lesions
Hwang et al. [24]	2021	177	58 (32-80)	142/35	Retrospective	HCC	Gadoxetic acid-enhanced MRI	-
Imbriaco et al. [25]	2017	73	60 ± 8.2	50/23	Prospective	HCC	Gadoxetic acid-enhanced MRI	-
Jang et al. [26]	2014	109	57.2 (26-79)	92/17	Retrospective	HCC	Gadoxetic acid-enhanced MRI	-
Kim et al. [27]	2006	31	57 (36-66)	28/3	Retrospective	HCC	Gadobenate dimeglumine-enhanced dynamic MRI	-
Min et al. [28]	2018	91	59 (32-76)	76/15	Prospective	HCC	Extracellular CE MRI	-
Min et al. [29]	2018	773	57.9 ± 10.1	612/161	Retrospective	HCC	Gadoxetic acid-enhanced MRI	-Capsule -Septum -T2 spotty hyperintensity
Min et al. [30]	2020	125	55.3 ± 8.8	102/23	Prospective	HCC	Extracellular CE MRI/gadoxetic acid-enhanced MRI	-
Min et al. [31]	2021	179	56.3 ± 8.6	145/34	Prospective	HCC	Extracellular CE MRI/gadoxetic acid-enhanced MRI	-
Morelli et al. [32]	2013	57	53.6 ± 14.5	26/31	Retrospective	HCC	Gadoxetic acid-enhanced MRI	-Enhancement ratio venous -Enhancement ratio hepatobiliary phase
Park et al. [33]	2021	294	56 ± 10	294/92	Retrospective	HCC	Gadoxetic acid-enhanced MRI	-
Rhee et al. [34]	2012	34	57 (30-66)	30/4	Retrospective	HCC	Gadoxetic acid-enhanced MRI	-Arterial enhancement -Washout -T1 hypointensity -T2 hyperintensity -Hepatobiliary hypointensity -Nodule size > 1.5 cm
Suh et al. [35]	2011	48	56.4 (20-85)	30/18	Retrospective	HCC	Gadoxetic acid-enhanced MRI	-Focal defect in uptake -Hypointense rim of hepatobiliary phase
Tsurusaki et al. [36]	2015	54	63 (35-84)	39/15	Prospective	HCC	Gadoxetic acid-enhanced MRI	-
Vandecaveye et al. [37]	2009	55	-	-	Prospective	HCC	DCE-MRI	-
Wei et al. [38]	2020	215	53.82 ± 11.24	166/49	Prospective	HCC	Gadoxetic acid-enhanced MRI	-Arterial phase hyperenhancement -Portal venous phase washout -Hepatobiliary phase hypointensity -Mild-moderate T2
Phongkitkarun et al. [39]	2013	100	59.5 ± 11.4	71/29	Retrospective	HCC	DCE-MRI	-
D’Onofrio et al. [40]	2014	149	59.4 (17-75)	93/54	Retrospective	HCA	Gadoxetic acid-enhanced MRI	-
Jansen et al. [41]	2019	95	-	-	Retrospective	HCA	DCE-MRI	-
Auer et al. [42]	2021	68	40 ± 12	9/59	Retrospective	HCA	Gadoxetic acid-enhanced MRI	-Gd-EOB uptake behavior -Gd-EOB uptake pattern -Arterial-phase hyperenhancement -Portal venous washout -Lobulated -Pseudocapsule -Central scar -Intralesional fat -Atoll sign
Reizine et al. [43]	2022	66	-	21/45	Retrospective	HCA	DCE-MRI	-
Cannella et al. [44]	2019	40	36.6 ± 9.5	1/39	Retrospective	HCA	Gadoxetic acid-enhanced MRI	-Hypointensity on portal venous phase imaging -Hypointensity on hepatobiliary phase imaging -Hyperintensity on T2-weighted imaging -Absence of central scar -Skewness on T2-weighted imaging -Skewness on HAP imaging -Entropy on HBP imaging -Skewness on HBP imaging
Grieser et al. [45]	2014	68	40.1 ± 10.5	5/63	Retrospective	HCA	Gadoxetic acid-enhanced MRI	-Arterial phase -Portal venous phase -Venous phase -Hepatobiliary phase
Zarghampour et al. [46]	2018	143	39.3 ± 8.5	52/91	Retrospective	HCA	DCE-MRI	-Hepatic arterial phase -Portal venous phase

Evaluation of the quality of research

Using a quality assessment tool, namely, QUADAS-2, we evaluated the quality of the studies. Eligible investigations were assessed in four main domains. The unclear risk of bias in the index text and reference standard was caused by not stating whether or not investigators were blinded when evaluating the index test or reference standard. The excluded studies were those with a high risk of bias in two or more domains, which depicts the results of quality assessment.

Hepatocellular carcinoma

**Figure 2:**
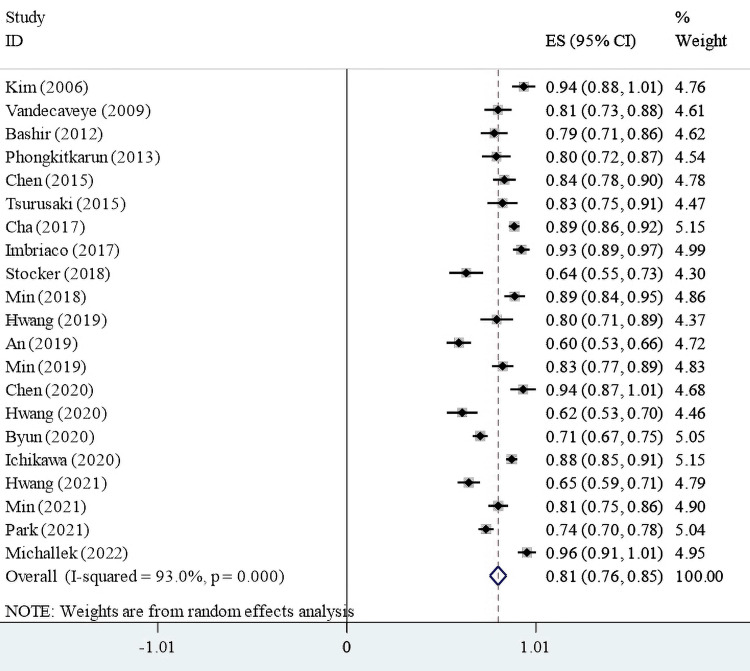
Forest plot for the diagnostic sensitivity of DCE-MRI to detect HCC tumors. DCE-MRI, dynamic contrast-enhanced magnetic resonance imaging; HCC, hepatocellular carcinoma

The overall DCE-MRI diagnostic accuracy and its features were pooled. Based on the random-effect model, the pooled sensitivity, specificity, accuracy, PPV, NPV, and AUC of DCE-MRI to distinguish HCC from other hepatocellular tumors were 80.6%, 88.2%, and 82.6%, respectively. The diagnostic accuracy of some DCE-MRI features was then pooled. The pooled sensitivity of T2 hyperintensity, hepatobiliary hypointensity, arterial enhancement, and portal-venous enhancement was 46.7% (95% CI = 16.4%-77.0%, *I*^2 ^= 99.2%, and *P *= 0.003), 86.8% (95% CI = 78.6%-94.9%, *I*^2 ^= 92.4%, and *P *< 0.001), 71.0% (95% CI = 54.6%-87.5%, *I*^2 ^= 95.9%, and *P *< 0.001), and 86.9% (95% CI = 82%-91.7%, *I*^2 ^= 64.8%, and *P *< 0.001), respectively. Besides, the pooled specificity of T2 hyperintensity, hepatobiliary hypointensity, arterial enhancement, and portal-venous enhancement was 88.3% (95% CI = 84.2%-92.3%, *I*^2 ^= 97.8%, *P *< 0.001), 79.9% (95% CI = 71.5%-88.3%, *I*^2 ^= 87.3%, *P *< 0.001), 77.7% (95% CI = 59.1%-96.3%, *I*^2 ^= 97.4%, and *P *< 0.001), and 53.6% (95% CI = 13.1%-94.1%, *I*^2 ^= 99.3%, and *P *= 0.01), respectively (Figure 2).

Hepatocellular adenoma

The pooled diagnostic accuracy of DCE-MRI and its features were calculated. Based on a random-effect model, the pooled sensitivity and specificity of DCE-MRI to differentiate HCA from other hepatocellular tumors were 86.2% (95% CI = 73.6%-98.8%, *I*^2^ = 95.5%, and *P* = 0.001) and 84.7% (95% CI = 71.8%-97.6%, *I*^2^ = 94.7%, and *P* = 0.001), respectively. Also, the features of the portal venous phase of DCE-MRI showed pooled sensitivity of 49.6% and pooled specificity of 76.7%, with 95% CI = 28.6%-70.6%, *I*^2^ = 95.8%, and 95% CI = 54.0%-99.4%, and *I*^2^ = 98.7%, respectively (*P *= 0.001). Furthermore, the arterial phase of DCE-MRI features had a pooled sensitivity of 77.3% (95% CI = 56.1%-98.5%, *I*^2^ = 95.9%, and *P* = 0.001) and pooled specificity of 47.0% (95% CI = 10.7%-83.3%, *I*^2^ = 98.0%, and *P* = 0.011).

Publication bias

**Figure 3:**
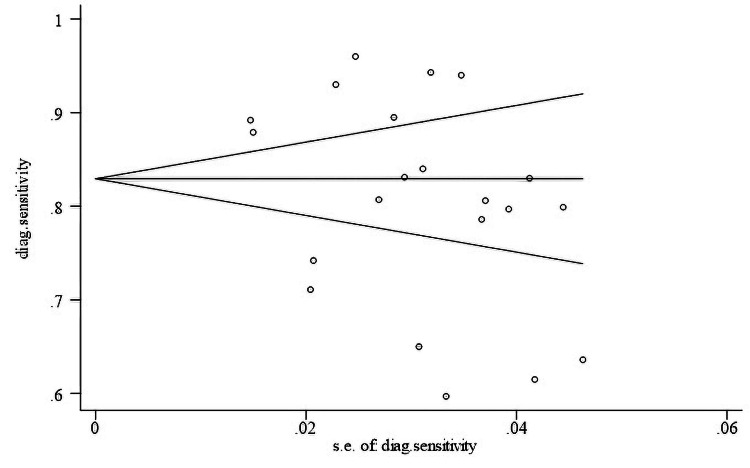
Publication bias assessment. Begg’s funnel plot with 95% confidence limits.

Egger's test and the funnel plot (p > 0.05), which were used to look at publication bias, showed that there was no obvious bias (Figure 3). 

Discussion

The present survey was undertaken to assess DCE-MRI diagnostic accuracy and its features to differentiate HCC and HCA from other hepatocellular tumors. Our results illustrated the pooled sensitivity of 80.6% and pooled specificity of 88.2% of DCE-MRI for HCC diagnosis. The pooled sensitivity and specificity of DCE-MRI for HCA diagnosis were 86.2% and 84.7%, respectively. Also, some DCE-MRI features, including T2 hyperintensity, hepatobiliary hypointensity, arterial enhancement, and portal venous enhancement, showed a pooled sensitivity of 46.7%, 86.8%, 71.0%, and 86.9%, respectively, and pooled specificity of 88.3%, 79.9%, 77.7%, and 53.6%, respectively, for HCC diagnosis using DCE-MRI. A reason for the heterogeneity of the included studies could be due to the different magnetic fields used in different studies. Moreover, some studies did not report a threshold and different thresholds may lead to heterogeneity.

Conventional ultrasonography has a major role in the HCC screening of at-risk patients [47]. Although US is cost-effective and widely available, its diagnostic accuracy in the detection of HCC is lower than that of contrast-enhanced CT and MRI [48,49]. Furthermore, HCC tumors 1 cm in size are isoechoic, making US difficult to detect [50]. A study revealed that the pooled sensitivity of US to detect HCC is 60% [51]. Despite the HCC, the pattern of HCA tumors in US is nonspecific. So, US cannot distinguish HCA from focal nodular hyperplasia (FNH). As a result, other imaging modalities, including dynamic contrast-enhanced CT, DWI, DCE-MRI, and CEUS, are used for HCC and HCA detection and grading [50,52,53]. During the process of carcinogenesis of the liver, the hemodynamic alterations occurring in the cirrhotic nodule while it progresses to early HCC are revealed in the dynamic contrast-enhanced CT. CEUS is another imaging modality and a decisive turning point in diagnosing HCC by sonography. CEUS features improved diagnostic accuracy for HCC detection and expanded CEUS's role in HCC diagnostic algorithms [50]. In addition, the function of CEUS features in detecting HCA has been illustrated [54]. However, there is not enough data on this emerging modality, and more studies can be conducted to reveal the effectiveness of CEUS [48]. The diagnostic tool diffusion-weighted imaging (DWI) is utilized to detect HCC tumors and evaluate HCC treatment responses. Changes in ADC values have been demonstrated to happen quickly after treatment and closely correlate with tumor necrosis [55]. Also, MRI is preferred to all other imaging modalities to detect HCA tumors and their subtypes [54]. It has been reported in guidelines that the recognition of a nodule in the liver of a patient by US should be followed by a dynamic MRI or CT [56]. However, due to the high diagnostic accuracy of DCE-MRI, some clinical guidelines for diagnosing HCC now include DCE-MRI as the first-line imaging method [14].

An earlier study, based on LI-RADS version 2018 and its modified version, assessed the diagnostic accuracy of DCE-MRI. The results exhibited sensitivities of 77% and 97%, specificities of 99% and 77%, and accuracies of 81% and 92%, according to LI-RADS version 2018 and modified LI-RADS IV (mLI-RADS), respectively [12]. Another study reported a sensitivity of 63.6% and specificity of 94.2% of DCE-MRI [17]. After assessing the diagnostic accuracy of some features of DCE-MRI, Chen et al. [57] stated that the sensitivity of the mean enhancement time (MET) was 58.8%, while the positive enhancement integral (PEI) and maximum slope of increase (MSI) were 70.6% and 82.4%, respectively. It has to be reported that, based on the result of this study, the specificity of all these features was 77%. Additionally, Mu et al. assessed the diagnostic value of hemodynamic parameters in DCE-MRI in HCC. Based on the results, the sensitivity of alpha-fetoprotein (AFP), volume transfer constant (*K*_trans_), and rate constant (*K*_ep_) were 88.9%, 86.7%, and 64.4%, respectively. Moreover, the specificities of AFP, *K*_trans_, and *K*_ep_ were 62%, 74%, and 90%, respectively. Our study had several limitations, as many studies were written in non-English languages. Also, the threshold was not stated in some articles, and different studies used different thresholds. Some studies did not reveal the criteria for HCC diagnosis that they used. And some unpublished studies were missed.

## Conclusions

In this review, the results of estimated sensitivity and specificity were satisfactory. Therefore, this strategy can serve as an appropriate tool for identifying HCC.
